# Establishment and Application of a Duplex TaqMan Real-Time PCR Assay for Simultaneous Detection of Chicken Anemia Virus (CAV) and *Gyrovirus Galga* 1 (GyVg1)

**DOI:** 10.3390/v18080859

**Published:** 2026-08-06

**Authors:** Kai-Xuan Gu, Zhi-Hao Ren, Xiao-Long Sun, Yang Li, Tao Sun, Wen-Ping Cui, Shuang Chang, Peng Zhao, Yu-Long Gao, Yi-Xin Wang

**Affiliations:** 1 College of Veterinary Medicine, Shandong Agricultural University, Tai’an 271018, China; 18363102597@163.com (K.-X.G.); zhren92@163.com (Z.-H.R.); sunxiaolong@sdau.edu.cn (X.-L.S.); sducwp@163.com (W.-P.C.); changshuang@sdau.edu.cn (S.C.); zhaopeng@sdau.edu.cn (P.Z.); 2 China Animal Health and Epidemiology Center, Qingdao 266032, China; liyang@cahec.cn; 3 Department of Health Management, Shandong Medicine Technician College, Tai’an 271001, China; 12626493@qq.com; 4 Harbin Veterinary Research Institute, Chinese Academy of Agricultural Sciences, Harbin 150069, China

**Keywords:** chicken anemia virus, Gyrovirus galga 1, duplex TaqMan real-time PCR, co-infection, sequence analysis

## Abstract

Both Chicken anemia virus (CAV) and *Gyrovirus galga* 1 (GyVg1) belong to the family *Anelloviridae* and genus *Gyrovirus*. They are non-enveloped, single-stranded circular DNA viruses that can infect chicken populations, leading to similar clinical symptoms such as growth retardation and immunosuppression, making clinical differentiation challenging. This study established a duplex TaqMan real-time PCR assay to simultaneously detect CAV and GyVg1. Specific primers and probes were developed to target the *VP3* gene of CAV (labeled with VIC) and the *VP1* gene of GyVg1 (labeled with FAM). The optimization process involved fine-tuning reaction conditions, such as primer/probe concentrations and annealing temperature, through a systematic matrix approach. The assay demonstrated remarkable linearity under optimized conditions (0.4 μmol/L primers, 0.2 μmol/L probes, and an annealing temperature of 52 °C), yielding high R^2^ values of 0.9973 for CAV and 0.9981 for GyVg1. The method exhibited high specificity, showing no cross-reactivity with other common avian pathogens. The limits of detection were 1.0 × 10^0^ copies/μL for CAV and 1.0 × 10^1^ copies/μL for GyVg1, making it 10-fold more sensitive than conventional PCR. Intra-assay and inter-assay coefficients of variation were below 1.5% and 2.5%, respectively, indicating excellent reproducibility. When tested on 411 clinical samples from Shandong Province, CAV showed a positivity rate of 48.66% (200/411), GyVg1 18.73% (77/411), and a co-detection rate of 17.52% (72/411). Moreover, 25 complete genome sequences of GyVg1 (2376 bp each) were acquired from positive samples. These sequences exhibited a nucleotide identity ranging from 93.6% to 99.3% among the isolates and 92.2% to 99.6% with reference strains. The phylogenetic analysis, which was based on whole-genome sequences, categorized the Shandong isolates into four clusters (I–IV). Notably, the strains were dispersed across these clusters and were intertwined with references from various regions and hosts. This observation suggests the absence of distinct geographic clustering and implies frequent cross-regional and cross-species transmission. In summary, this duplex real-time PCR assay offers a sensitive, specific, and dependable method for concurrently detecting and distinguishing CAV and GyVg1. The study’s findings reveal a significant co-infection rate and genetic variation in GyVg1, underscoring the importance of increased surveillance and deeper exploration of its pathogenicity and transmission patterns.

## 1. Introduction

Gyroviruses, initially placed in the *Circoviridae* family, have been reclassified under the family *Anelloviridae* due to their genomic structure and genetic features [1]. These viruses belong to the group of non-enveloped, single-stranded circular DNA viruses [2]. Chicken anemia virus (CAV) is the prototype member of this group and has been a focal point as a crucial immunosuppressive pathogen in poultry for a long time [3,4,5]. The increasing accessibility of high-throughput sequencing technologies has led to the successive discovery of numerous novel gyroviruses in avian species and environmental samples worldwide [6,7], significantly broadening our knowledge of the diversity and host range of this viral group. *Gyrovirus galga* 1 (GyVg1, previously known as avian gyrovirus 2, AGV2) has swiftly emerged as a key focus in avian disease research, owing to its extensive host range and potential for inter-species transmission. Both CAV and GyVg1 are classified under the *Gyrovirus* genus, primarily affecting chickens [8], and are prevalent in China and worldwide, posing an enduring threat to poultry health.

The genome of CAV comprises a single-stranded negative-sense circular DNA of approximately 2.3 kb [9,10]. This DNA contains three partially overlapping open reading frames (ORFs) that encode the capsid protein VP1, the non-structural protein VP2, and the pathogenic protein VP3 (apoptin). VP1 serves as the exclusive structural protein of the virus, triggering the production of neutralizing antibodies in the host. Meanwhile, VP2 functions as a scaffold protein in the viral assembly process. Additionally, VP3 is responsible for initiating apoptosis in lymphoid cells and erythroid progenitor cells, resulting in organ damage [11]. CAV predominantly impacts young chicks, particularly those without maternal antibodies. The virus leads to severe aplastic anemia and thymic atrophy by targeting hematopoietic stem cells in the bone marrow and T-lymphocyte precursor cells in the thymus. Clinically, it appears as anemia, hemorrhages, growth retardation, and immunosuppression [11,12]. These symptoms not only increase mortality directly but also exacerbate secondary infections due to immune failure, resulting in significant economic losses for the poultry industry [13,14].

The knowledge about GyVg1 is relatively rudimentary when contrasted with CAV. Discovered in Brazilian chicken flocks in 2011, GyVg1 exhibits a genomic structure closely resembling that of CAV [15]. Both viruses consist of a single-stranded circular DNA of around 2.3–2.4 kb, which encodes the proteins VP1, VP2, and VP3 [16]. Nevertheless, GyVg1 displays a remarkably wide host range. Apart from poultry such as chickens [16,17], peafowl [18], and pheasants, it has been frequently identified in various mammalian species like dogs, cats, ferrets, hippopotamuses, tigers, lions, and sika deer, and has even been found in human samples [18,19]. This indicates a significant potential for cross-species transmission. Epidemiological studies have revealed that GyVg1 is prevalent in poultry across multiple continents, spanning Asia [16,20], Europe, South America, and Africa [20]. The pathogenicity of GyVg1 remains incompletely understood [17]; however, several studies have linked it to clinical manifestations like growth retardation, weakness, and diarrhea in chicken flocks. The contamination incident in Brazil, where GyVg1 was detected in 9 out of 35 vaccines tested, has sparked concerns in the industry regarding the safety of biological products. Given that GyVg1 is commonly found in co-infections with other pathogens, its specific role as a primary pathogen necessitates further investigation.

Due to significant similarities and genetic overlap in their background, host range, and clinical signs, distinguishing between CAV and GyVg1 based on clinical presentation is highly challenging. Therefore, it is imperative to establish a diagnostic method capable of simultaneously, rapidly, and accurately detecting and differentiating these two viruses. This is crucial for clinical differentiation, epidemiological surveillance, and the development of prevention and control strategies. Conventional methods like virus isolation or singleplex PCR have limitations such as complex procedures, low sensitivity, and inadequate capacity, which are not sufficient for detecting mixed infections effectively. In contrast, the duplex TaqMan real-time PCR technology can identify two targets simultaneously in a single reaction, providing high sensitivity, specificity, and quantitative capabilities [21,22].

Previously, Varela et al. developed a duplex quantitative real-time PCR (dqPCR) capable of detecting CAV and AGV2 (now GyVg1) genomes in poultry vaccines, primarily for contamination screening [23]. However, a duplex TaqMan real-time PCR assay specifically targeting the *VP3* gene of CAV and the *VP1* gene of GyVg1, with systematic optimization for clinical sample detection and large-scale epidemiological application, has not been well established. Therefore, this study aims to develop a duplex TaqMan real-time PCR method for detecting CAV and GyVg1 simultaneously, optimize the reaction system, and assess its performance systematically. Moreover, this approach will be utilized to examine clinical samples in order to determine the prevalence and co-infection patterns of the two viruses in chicken populations in Shandong Province. Concurrently, GyVg1-positive samples will undergo whole-genome amplification and sequence analysis to investigate the genetic variability and evolutionary traits of the prevalent strains in this area. This research will establish a dependable technical method for promptly distinguishing between CAV and GyVg1 and will lay the groundwork for a more profound comprehension of the molecular epidemiology and inter-species transmission dynamics of GyVg1. Consequently, this will facilitate the precise prevention and management of avian diseases.

## 2. Materials and Methods

### 2.1. Primer Design

Based on the gene sequences of CAV (GenBank accession numbers: AF313470, M55918, D10068) and GyVg1 (GenBank accession numbers: JQ690763, OK245349M, KX708518) published in GenBank, their conserved sequences were analyzed using MegAlign (version 17.6) software (DNAStar Inc., Madison, WI, USA). Fluorescence-specific primers and a TaqMan probe for GyVg1 were designed using Primer Premier 5.0 software. The primers and probes for GyVg1 are designed in the conserved region of the *VP1* gene, while those for CAV are designed in the conserved region of the *VP3* gene. The target genes were selected based on their distinct biological characteristics and sequence conservation. For CAV, the *VP3* gene was chosen because it is highly conserved across CAV strains and is directly associated with the virus’s pathogenic effects, making it a reliable diagnostic marker. For GyVg1, the *VP1* gene was selected because it contains conserved regions suitable for primer/probe design, and it provided the most reliable sequence data among the limited available GyVg1 genomes. The primer and probe sequence information is presented in Table 1. The primers and probe were synthesized by Sangon Biotech (Shanghai, China).

### 2.2. Screening for the Optimal Reaction System in Duplex Real-Time PCR

DNA was extracted from clinical samples positive for CAV and GyVg1 using a viral DNA extraction kit (Tiangen Biotech, Beijing, China, Cat. No. DP315), following the manufacturer’s instructions. The duplex real-time PCR was performed on a Real-Time Fluorescent Quantitative PCR System (Archimed X6, Rocgene, Beijing, China). The real-time PCR reaction system was optimized for template amount, probe concentration, primer concentration, and annealing temperature through a matrix approach. Primer concentrations ranged from 0.1 to 1.0 μmol/L, and probe concentrations ranged from 0.05 to 0.5 μmol/L. Evaluation criteria were based on achieving the lowest Ct value and the highest fluorescence intensity (ΔRn). The reaction system proceeded as follows: 10 μL of 2× Premix Ex Taq (Probe qPCR) (TaKaRa, Dalian, China, Cat. No. RR390A), along with 2 μL of template, primers, and probe, were combined, and the volume was adjusted to 20 μL with ddH_2_O. The real-time PCR reaction program consisted of an initial pre-denaturation at 95 °C for 5 min, followed by 40 cycles of denaturation at 95 °C for 15 s and annealing at five different temperatures (50, 52, 54, 56, and 58 °C) for 1 min. The probe concentration, primer concentration, and annealing temperature associated with the minimum threshold cycle (Ct) value were carefully chosen.

### 2.3. Establishment of the Duplex Real-Time PCR Standard Curve

To establish plasmid standards, the *VP1* gene of GyVg1 and the *VP3* gene of CAV were amplified through PCR. The PCR reaction mixture, totaling 50 μL, included 25 μL of Premix Taq master mix, 2 μL each of forward and reverse primers, 4 μL of DNA template, and 6 μL of ddH_2_O. The PCR process involved an initial denaturation step at 95 °C for 3 min, followed by 35 cycles of denaturation at 95 °C for 30 s, annealing at 56 °C for 30 s, and extension at 72 °C for 1 min. A final extension step at 72 °C for 2 min concluded the PCR. Subsequently, the PCR products were purified and inserted into the pMD18-T vector to create the standard plasmids pMD-GyVg1 and pMD-CAV. The plasmid concentrations were determined, and the copy numbers were calculated using the formula: copy number per μL = [nucleic acid concentration (ng/μL) × 6.022 × 10^23^]/[nucleic acid length (bp) × 10^9^ × 650]. The plasmids were adjusted uniformly to a working concentration of 10^8^ copies/μL. Subsequently, a 10-fold serial dilution series comprising nine gradients was prepared for each plasmid, and each dilution was utilized as a template, with a negative control set concurrently. Amplification was carried out using the optimized reaction system and conditions of the duplex real-time PCR method. The standard curve was then established by plotting the logarithm of the initial copy number of the standard against the resulting Ct value.

### 2.4. Specificity, Sensitivity, and Reproducibility Validation

The optimized duplex real-time PCR method was employed to analyze DNA or reverse-transcribed cDNA from CAV, GyVg1, MDV, FPV, REV, ALV-A/B/J/K, H9N2 AIV, NDV, IBV, aMPV, MG, and MS. Plasmid standards served as target templates, and a negative control was included simultaneously to confirm the specificity of the method. For the sensitivity test, the plasmid standards underwent 10-fold serial dilutions, with each dilution serving as a template. The sensitivity was evaluated using the established method, and conventional PCR amplification and DNA electrophoresis were carried out simultaneously. A negative control was also included for comparison. The limits of detection of both methods were then compared and assessed. To assess reproducibility, three consecutive concentration gradients of plasmid standards were randomly chosen after gradient dilution. These gradients were split into two batches, with each concentration tested in triplicate to assess intra-assay reproducibility. For inter-assay reproducibility, the same samples underwent amplification using the optimized duplex real-time PCR method three days later. The coefficient of variation (CV) was then calculated based on the Ct values obtained to confirm the method’s reproducibility.

### 2.5. Detection of Clinical Samples and Whole-Genome Cloning and Sequencing of GyVg1

In 2024, a total of 411 clinical samples were collected across 12 cities in Shandong Province, China. The samples comprised 312 cloacal swabs and 99 tissue samples, including thymus (*n* = 42), bursa of Fabricius (*n* = 35), and bone marrow (*n* = 22). All birds exhibited clinical signs consistent with infectious anemia or were from flocks with suspected immunosuppression, including growth retardation, depression, pale combs/wattles, and increased mortality. None of the sampled flocks had been vaccinated against CAV. A detailed breakdown of sample distribution by city and sample type is provided in Supplementary Table S1. The detection of CAV and GyVg1 was performed through duplex real-time PCR and conventional PCR, and the consistency between the two methods was assessed. To validate the discordant results between the two methods, all samples that tested positive by duplex real-time PCR but negative by conventional PCR were subjected to nested PCR amplification using the same primer sets targeting the *VP3* gene of CAV and the *VP1* gene of GyVg1. The amplified products were subsequently purified and sequenced by Sanger sequencing. In order to analyze the genetic differentiation characteristics of GyVg1 in specific regions of Shandong Province, 25 positive samples were chosen. Three pairs of primers (Table 1) were employed to amplify the entire coding region of the virus. The PCR reaction system was implemented, and the PCR products underwent 1% agarose gel electrophoresis and were then retrieved using a gel extraction kit (Omega, Guangzhou, China). The purified products were subsequently cloned into the pMD18-T vector for further cloning and sequencing.

### 2.6. Sequence Analysis

Sequence assembly was carried out by utilizing the SeqMan program in the DNASTAR Lasergene software package (version 17.6) (DNAStar Inc., Madison, WI, USA). The 25 sequences acquired were submitted to the GenBank database with accession numbers PX418107–PX418130. A phylogenetic tree of the full-length *VP1* gene was created using the Neighbor-Joining method with the Kimura 2-parameter model and 1000 bootstrap replicates. Sequence alignment was conducted using Clustal W, and genetic distances were computed using MEGA 11 software. The reference sequence information is presented in Table 2. Meanwhile, the VP1 proteins of 25 field isolates were aligned, and the differential amino acids at key sites were screened and analyzed.

## 3. Results

### 3.1. Establishment and Optimization of the Duplex Real-Time PCR System

The optimal concentrations of primers and probes for GyVg1 and CAV were determined through a matrix approach. The findings indicated that using GyVg1 primer concentration of 0.4 μmol/L and probe concentration of 0.2 μmol/L, along with CAV primer concentration of 0.4 μmol/L and probe concentration of 0.2 μmol/L, resulted in the lowest Ct values (GyVg1: 21.35 ± 0.12; CAV: 22.01 ± 0.09) and the most intense fluorescence signals for both viruses at standards of 1 × 10^5^ copies/μL. These concentrations also showed no significant interference between the fluorophores (FAM and VIC) of the two probes in the duplex reaction system. With the optimal primer and probe concentrations, qPCR amplification was conducted at annealing temperature gradients ranging from 56 °C to 64 °C. The findings indicated that the Ct values for GyVg1 and CAV were minimized at an annealing temperature of 52 °C. Consequently, 52 °C was identified as the optimal annealing temperature.

### 3.2. Construction of the Duplex Real-Time PCR Standard Curve

**Figure 1:**
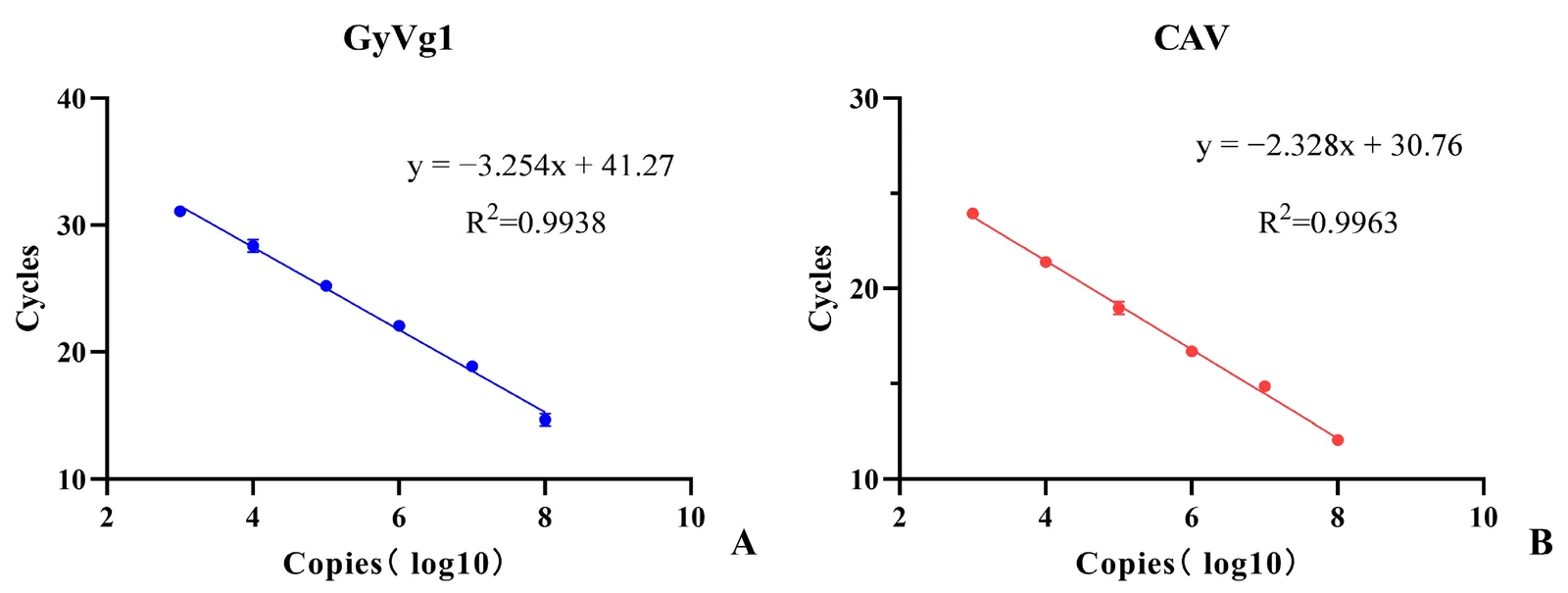
Establishment of standard curves for the duplex real-time fluorescent quantitative PCR for GyVg1 and CAV: (**A**) Standard curve for GyVg1. (**B**) Standard curve for CAV.

The plasmid standards pMD-CAV and pMD-GyVg1, with concentrations ranging from 1.0 × 10^8^ to 1.0 × 10^1^ copies/μL, served as templates. Each copy number was utilized for amplification in the optimized duplex real-time PCR system. The standard curve was created by plotting the Ct value against the common logarithm of the template copy number (lg copies) (Figure 1). For GyVg1, the standard curve equation was y = −3.254x + 41.27, with a correlation coefficient R^2^ = 0.9938. Concerning CAV, the standard curve equation was y = −2.328x + 30.76, yielding a correlation coefficient R^2^ = 0.9963. These findings demonstrate a high level of linearity and efficient amplification across a wide dynamic range, fulfilling the criteria for precise quantification.

### 3.3. Specificity Test of Duplex Real-Time PCR Assay

**Figure 2:**
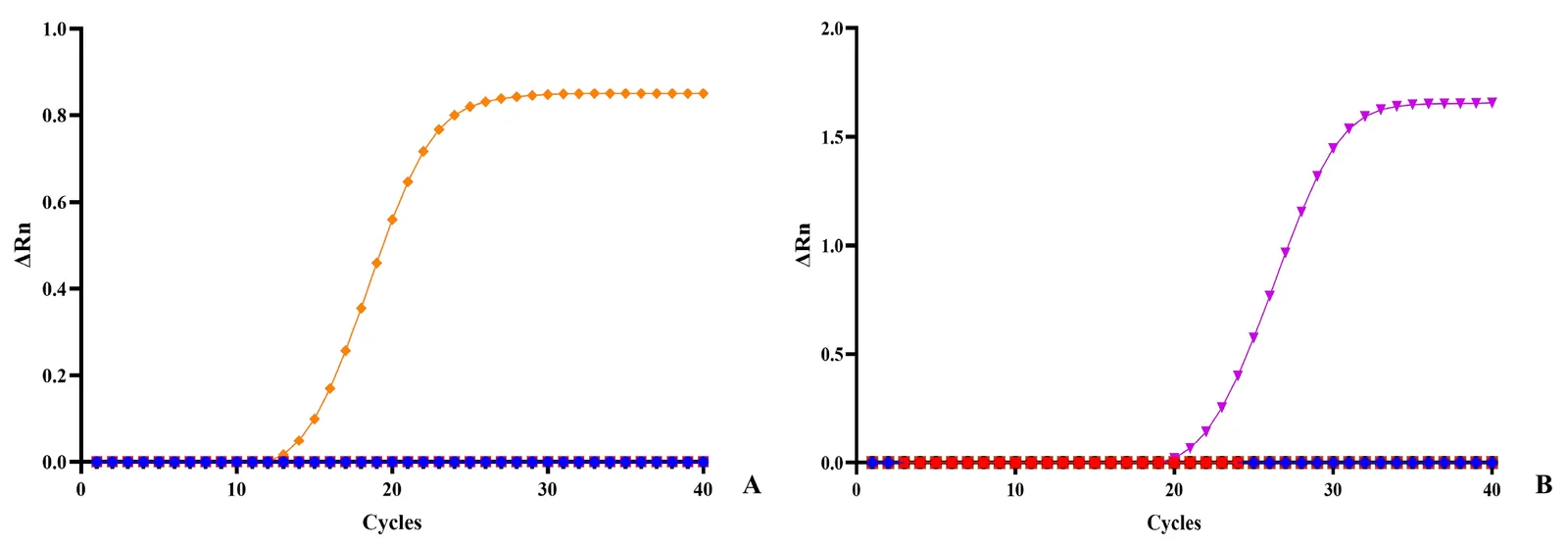
Specificity test of the duplex real-time PCR for GyVg1 and CAV: (**A**) Specificity test for GyVg1 detection. (**B**) Specificity test for CAV detection.

DNA samples containing a mixture of GyVg1 and CAV, and nuclease-free water were utilized as templates for amplification in the duplex TaqMan real-time PCR system. The findings revealed that the GyVg1 template generated a distinct amplification curve solely in the FAM channel, while the CAV template produced an amplification curve exclusively in the VIC channel (Figure 2). Notably, no amplification curve was detected in the negative control. In order to further confirm the specificity of the method, nucleic acids from common avian pathogens were utilized as templates, while GyVg1 and CAV positive controls were concurrently established. The findings revealed that none of the non-target pathogens exhibited distinct fluorescence signals, whereas all positive controls yielded typical amplification curves. These outcomes validate the high specificity of this method for GyVg1 and CAV, as there was no cross-reactivity with other avian pathogens, underscoring the robust specificity of the established duplex real-time PCR detection technique.

### 3.4. Sensitivity Test of Duplex Real-Time PCR Assay

**Figure 3:**
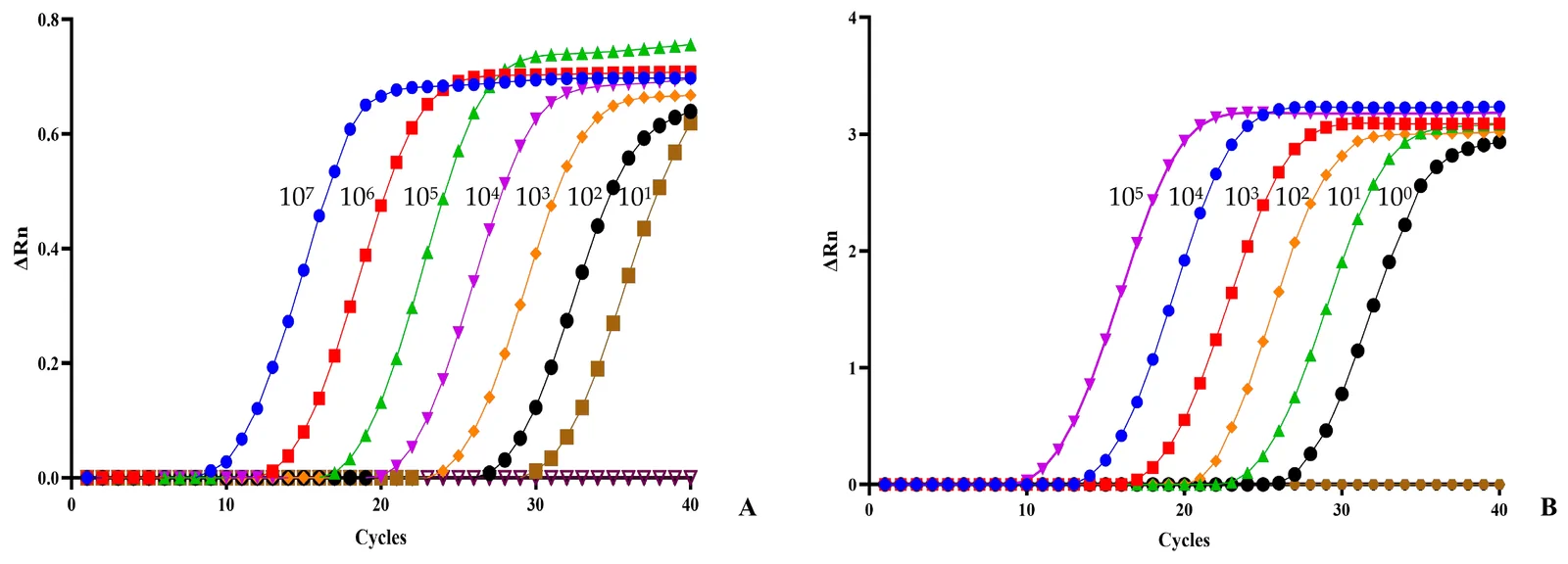
Sensitivity test of the duplex real-time PCR for GyVg1 and CAV: (**A**) Sensitivity test for GyVg1 detection. (**B**) Sensitivity test for CAV detection.

**Figure 4:**
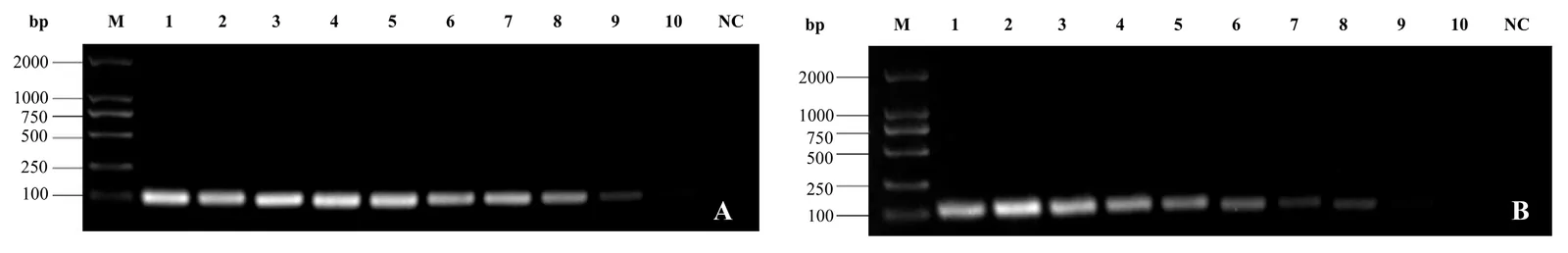
Sensitivity test of conventional PCR: (**A**) Sensitivity test for GyVg1 detection. Lane M, DNA marker; lanes 1–10, 10-fold serial dilutions of pMD-GyVg1 from 10^9^ to 10^0^ copies/μL; NC, negative control. (**B**) Sensitivity test for CAV detection. Lane M, DNA marker; lanes 1–10, 10-fold serial dilutions of pMD-CAV from 10^9^ to 10^0^ copies/μL; NC, negative control.

The GyVg1 and CAV recombinant plasmid standards underwent 10-fold serial dilutions ranging from 1 × 10^7^ to 1 × 10^0^ copies/μL. Subsequently, duplex TaqMan real-time PCR was employed to compare the detection limits in both scenarios. The analysis demonstrated that the duplex real-time PCR method could detect CAV and GyVg1 at limits of 1.0 × 10^0^ and 1.0 × 10^1^ copies/μL, respectively (Figure 3). Conversely, conventional PCR showed limits of detection for CAV and GyVg1 at 1.0 × 10^1^ and 1.0 × 10^2^ copies/μL, respectively (Figure 4). These findings underscore the heightened sensitivity of the duplex real-time PCR method devised in this research.

### 3.5. Reproducibility Test of Duplex Real-Time PCR Assay

The templates for pMD-CAV and pMD-GyVg1 were chosen at concentrations of 1.0 × 10^7^, 1.0 × 10^5^, and 1.0 × 10^3^ copies/μL, and each was analyzed in triplicate within the same batch. Mean Ct value, standard deviation (SD), and coefficient of variation (CV) were computed. The findings revealed that GyVg1’s intra-assay CV ranged from 0.56% to 1.02%, and CAV’s ranged from 0.48% to 0.95%, all below 1.5%, indicating excellent intra-assay reproducibility of the method (Table 3).

### 3.6. Clinical Sample Testing

A total of 411 clinical samples suspected of being infected with CAV or GyVg1 underwent testing using a duplex real-time PCR method and were simultaneously subjected to conventional PCR analysis. As shown in Table 4, the results indicate that the duplex real-time PCR method yielded a positive rate of 48.66% (200/411) for CAV, 18.73% (77/411) for GyVg1, and a co-infection detection rate of 17.52% (72/411). In contrast, conventional PCR showed positive rates of 39.41% (162/411) for CAV, 15.09% (62/411) for GyVg1, and a co-infection detection rate of 13.14% (54/411). The overall concordance rate for CAV is 90.75%, while for GyVg1, it is 96.35%. Sequencing verification of the 38 discordant samples confirmed that all samples were true positives. Both show high overall consistency, with all conventional PCR positives being covered by real-time PCR, indicating that the dual real-time PCR method has a higher detection efficiency. The 95% confidence intervals (CIs) for the detection rates were calculated as follows: 48.66% (95% CI: 43.82–53.50%) for CAV, 18.73% (95% CI: 15.08–22.38%) for GyVg1, and 17.52% (95% CI: 13.86–21.18%) for co-detection. To compare the performance of duplex real-time PCR and conventional PCR, McNemar’s test was applied. The duplex real-time PCR detected significantly more CAV-positive samples than conventional PCR (*p* < 0.001) and also detected significantly more GyVg1-positive samples (*p* = 0.008), indicating superior sensitivity of the established method.

### 3.7. Analysis of the Complete Genome Sequences of GyVg1

Four pairs of overlapping primers were utilized to amplify GyVg1’s complete genome from real-time PCR-positive samples. The PCR products underwent clone sequencing and assembly, resulting in 25 successful GyVg1 genome sequences. These sequences were officially deposited in GenBank with accession numbers PX418107-PX418130, and detailed information is available in Table 5. All sequences maintained a consistent length of 2376 bp, showing no insertions or deletions when compared with the reference sequence. The genome structure exhibited the typical features of GyVg1, comprising three primary ORFs responsible for encoding VP1 (capsid protein), VP2 (scaffold protein), and VP3 (apoptin), along with a non-coding region positioned between these ORFs. Pairwise comparison of the 25 complete GyVg1 genome sequences showed nucleotide homology ranging from 93.6% to 99.3%. The strains SD2024-10 and SD2024-11 had the lowest homology at 93.6%, while the highest homology of 99.3% was found between SD2024-7 and SD2024-9. In comparison with other reference strains, the nucleotide homology varied from 92.2% to 99.6%. Strain SD2024-23 exhibited the lowest homology at 92.2% with G17, whereas the highest homology of 99.6% was observed between SD2024-9 and GS1512.

**Figure 5:**
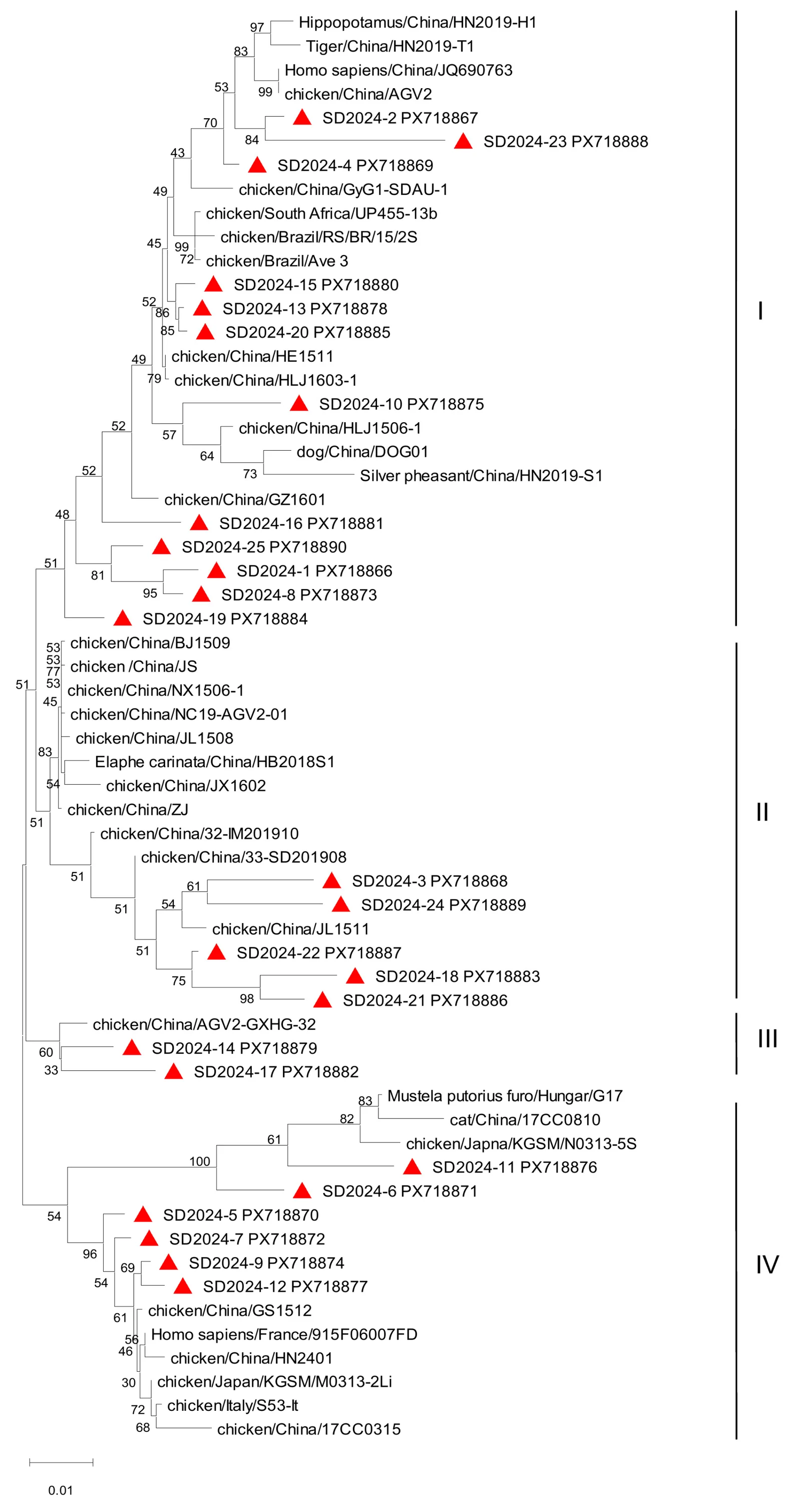
Phylogenetic analysis of GyVg1 based on complete genome sequences. Based on the *VP1* gene sequences, GyVg1 isolates were classified into four distinct clusters, designated I, II, III, and IV. The red triangle symbols in the figure represent the viral strains identified in this study.3.8. Analysis of the VP1 Amino Acid Sequences of GyVg1.

In order to examine the evolutionary relationships between the 25 GyVg1 strains identified in this study and other reference strains, a phylogenetic tree was developed using the complete genome sequences of these strains (Figure 5). The analysis of the complete genomes of the 25 GyVg1 strains demonstrated a varied distribution pattern for the Shandong strains, indicating the concurrent circulation of multiple GyVg1 genotypes in Shandong Province. The analysis revealed that the phylogenetic tree could be categorized into four clusters: Cluster I, comprising 12 strains; Cluster II, comprising 5 strains; Cluster III, comprising 2 strains; and Cluster IV, comprising 6 strains. Notably, Cluster I had the largest number of strains, indicating its status as the predominant circulating lineage. Additionally, in the phylogenetic tree, Cluster IV displayed a relatively distant relationship with the other clusters, potentially signifying an independent evolutionary branch or a novel subtype. The Shandong isolates were spread out among all clusters and grouped together with reference strains, rather than forming a distinct geographic branch. This suggests a potential cross-regional transmission of the virus. GyVg1 strains from dogs, cats, and wild animals were found in the same evolutionary branches as GyVg1 strains from chickens, indicating a broad host adaptability or possible genetic recombination.

To investigate variations in the main structural protein, amino acid sequence alignment of the VP1 capsid protein was performed for 25 field isolates recovered in this study, together with three reference strains: AGV2-GXHG-32/China (OR269224), AGV2/China (KX870138), and Ave_3/Brazil (KX870137). Analysis of 17 informative residues revealed that the isolates in our study shared a highly conserved backbone with the Chinese reference strain AGV2/China, yet exhibited characteristic mutations at specific loci previously identified as hypervariable or under positive selection (Table 6). At residues 36, 73, 106, 212, 242, 270, 390, 401, 416, 433, and 459, all 25 isolates displayed complete conservation with AGV2/China (G, R, L, S, K, R, S, I, V, L, W, and N, respectively). Notably, residues 212 (Lys), 270 (Ser), and 310 (Glu) were invariant across the entire panel, consistent with their designation as structurally or functionally critical sites in GyVg1 VP1. Similarly, position 44 was uniformly arginine (R) in all isolates, matching AGV2/China and Ave_3/Brazil but differing from AGV2-GXHG-32, which carries a lysine (K) at this site. The most prominent polymorphism was observed within the central hypervariable region spanning residues 288–293. Two distinct haplotypes co-circulated among the isolates: fifteen strains (60%) harbored valine at position 288 and glycine at position 293 (V288/G293), identical to the pattern seen in AGV2-GXHG-32 and Ave_3/Brazil; whereas ten strains (40%) possessed glutamine at both positions (Q288/Q293), matching the AGV2/China reference. Additional sporadic mutations were detected at positions 314 and 383. Four isolates (SD2024-5, -6, -11, and -12) exhibited a lysine (K) substitution at residue 314 instead of the consensus arginine (R) conserved across all reference strains. Furthermore, one isolate (SD2024-23) carried a serine (S) at position 383, diverging from the proline (P) present in AGV2/China and AGV2-GXHG-32. No substitutions were observed at residues 12, 114, 123, 167, 231, 237, 243, 335, or 444, which have been associated with host adaptation in companion-animal–derived GyVg1 strains.

## 4. Discussion

Both CAV and GyVg1 are part of the *Gyrovirus* genus [3,15]. They have similar genomic structures and each contains three partially overlapping open reading frames: VP1, VP2, and VP3 [24]. These viruses can both infect chicken flocks, leading to clinical presentations with overlapping features [11,16,25]. These symptoms, such as poor growth and depression, can arise from single-virus infection, mixed infection, or combined effects with other pathogens. Consequently, it is crucial to devise a method that can rapidly and accurately detect and differentiate these two viruses simultaneously. The duplex TaqMan real-time PCR method, as established in this study, demonstrated exceptional performance across several critical indicators. The method exhibited high specificity for CAV and GyVg1, without any cross-reactivity with other common avian pathogens. The limit of detection for CAV was 1.0 × 10^0^ copies/μL, and for GyVg1, it was 1.0 × 10^1^ copies/μL, showing significantly improved sensitivity compared with conventional PCR. The intra-assay CVs were all below 1.5%, and the inter-assay CVs were all below 2.5%, indicating the method’s good stability and reproducibility. In contrast to traditional virus isolation or singleplex PCR detection, this method can concurrently identify two targets in a single reaction system, thereby enhancing detection efficiency and reducing time and costs. The results’ accuracy is ensured by both the high sensitivity and high specificity, along with the quantitative capability that provides precise information on viral load in samples. This, in turn, facilitates epidemiological screening and disease surveillance for large-scale clinical samples.

Although Varela et al. [23] previously developed a duplex qPCR for CAV and AGV2 (now GyVg1) primarily for vaccine contamination screening, that study focused primarily on vaccine screening and did not systematically optimize reaction conditions for clinical samples. Compared with these previous methods, our assay specifically targets the *VP3* gene of CAV and the *VP1* gene of GyVg1, with optimized primer/probe concentrations and annealing temperature through a matrix approach, achieving detection limits of 1 and 10 copies/μL for CAV and GyVg1, respectively. Moreover, our method was systematically validated with a broad panel of avian pathogens and applied to a large clinical cohort in Shandong Province, demonstrating its robustness for routine diagnostics and epidemiological surveillance. Regarding GyVg1 detection methods, previous studies have employed conventional PCR [16,17], nested PCR, or singleplex real-time PCR for GyVg1 identification. For instance, Tran et al. [17] detected GyVg1 in Vietnamese chickens using conventional PCR with a positivity rate of 20.63%. Zhang et al. [16] applied a singleplex qPCR for GyVg1 surveillance in southern China. Our duplex TaqMan method not only achieves comparable or higher sensitivity (10 copies/μL) but also enables simultaneous detection and differentiation of CAV and GyVg1 in a single reaction, significantly improving diagnostic efficiency for co-infection screening. The ability to quantify viral loads further provides an advantage over conventional gel-based methods for monitoring disease dynamics and viral shedding.

The established method was utilized to examine 411 clinical samples suspected of infectious anemia from various regions in Shandong Province. The findings indicated a CAV positive rate of 48.66% (200/411), a GyVg1 positive rate of 18.73% (77/411), and a co-detection rate of 17.52% (72/411). These outcomes are in line with previous research findings. CAV, a widely distributed immunosuppressive pathogen, typically shows a high positivity rate in clinical samples [4]. The 48.66% positivity rate in this study further validates the extensive prevalence of CAV in chicken flocks in China [9,26]. It is noteworthy that the GyVg1 positivity rate (18.73%) in this study aligns with findings from other regions of China in recent years [27]. Between 2021 and 2024, a study revealed a significant GyVg1 detection rate in 63 poultry farms across six provinces in central and eastern China, namely Jiangsu, Anhui, Henan, Hunan, Shandong, and Hubei. The detection rate ranged from 11.69% in Guangdong to 22.46% in Yunnan [16]. These findings suggest that GyVg1 is already prevalent in chicken flocks across various regions in China.

It is noteworthy that this study revealed a co-detection rate of CAV and GyVg1 at 17.52% (72/411), indicating a possible common co-infection in chicken flocks. While mixed infections within the *Gyrovirus* genus have been documented, the high co-detection rate observed in this study raises the possibility of synergistic pathogenic effects, which could potentially worsen immunosuppression and elevate the risk of secondary infections [8,28]. Both CAV and GyVg1 are part of the Gyrovirus genus and exhibit significant resemblance in genomic structure, coding strategy, and pathogenic mechanism. The VP3 protein (apoptin) found in CAV has the ability to trigger apoptosis in infected lymphoid cells and erythroid progenitor cells. This process leads to thymic atrophy and aplastic anemia, ultimately causing severe immunosuppression [11,29]. The genetic makeup of GyVg1 includes the genes for VP1, VP2, and VP3, resembling that of CAV closely [30,31]. Despite these similarities, the full extent of its pathogenicity remains unclear. GyVg1 has been linked to symptoms like stunted growth, weakness, and diarrhea in poultry, yet its specific role in causing disease warrants further investigation due to its frequent co-occurrence with other pathogens. Experimental infection studies and re-isolation trials are needed to confirm GyVg1’s independent pathogenic potential. The similarity in host target cells affected by both viruses may result in comparable immunopathological damage. CAV mainly targets hematopoietic stem cells in the bone marrow and T-lymphocyte precursors in the thymus, while it remains uncertain if GyVg1 exhibits the same cell tropism [21,32]. Further confirmation is needed through artificial infection trials and pathogenicity investigations to determine whether GyVg1 functions as a primary pathogen or an opportunistic virus in chickens.

This study successfully acquired 25 complete genome sequences of GyVg1 in various parts of Shandong Province. All sequences were 2376 bp long, maintaining the typical genomic characteristics of GyVg1 without any insertions or deletions compared with the reference sequence. Analysis of sequence homology indicated that nucleotide homology among the 25 Shandong isolates ranged from 93.6% to 99.3%, while homology with reference strains varied from 92.2% to 99.6%. Phylogenetic analysis based on the complete genome displayed a diverse distribution pattern for the 25 Shandong isolates, leading to their classification into four distinct phylogenetic clusters (I–IV). Cluster I comprised 12 strains and represented the predominant circulating lineage. Notably, the Shandong isolates were evenly grouped with reference strains from various regions and hosts, rather than forming a distinct geographic branch. This aligns with other findings, indicating no clear link between GyVg1 evolutionary branches and their geographical distribution [16]. This trend implies potential frequent cross-regional transmission of GyVg1. It is noteworthy that GyVg1 strains from dogs, cats, and wild animals were dispersed across the same evolutionary branches as chicken-derived strains in this study [18]. This suggests broad host adaptability of GyVg1, potentially indicating cross-species transmission and genetic recombination [30]. GyVg1 was initially identified in diseased chickens in Brazil in 2011 and has since been found in various mammals, such as humans, ferrets, domestic cats, domestic dogs, hippopotamuses, tigers, lions, sika deer, and other species [16,18,33]. Both this research and other studies have indicated that GyVg1 might undergo cross-species recombination events [17]. This broad host range and potential for cross-species transmission complicate the evolution and epidemiology of GyVg1 [18,34]. While there is currently insufficient evidence to confirm GyVg1’s pathogenicity in mammals, including humans, its widespread presence in multiple hosts implies that the virus possesses significant host adaptability and potential public health implications [6,33].

The VP1 protein of GyVg1 constitutes the major structural component of the viral capsid and is believed to mediate host receptor binding and immune recognition [35]. In the present study, the 25 isolates displayed a largely conserved VP1 sequence when compared with the prototype AGV2/China strain, with the majority of variation concentrated within the three previously defined hypervariable regions: the N-terminal domain (residues 44–73), the central region (288–316), and the C-terminal cluster (383–419) [16,30]. This spatial restriction of mutations supports the hypothesis that VP1 evolves under strong structural constraints, wherein surface-exposed loops tolerate amino acid substitutions while core residues essential for capsid integrity remain invariant. The strict conservation of residues 212 (Lys), 270 (Ser), and 310 (Glu/Gln) across all isolates and reference strains underscores their likely importance for protein folding or virion assembly. Zhang et al. [30] previously noted that these positions are refractory to mutation even in large-scale surveillance datasets, suggesting they may participate in critical intra- or inter-molecular interactions. By contrast, the bifurcated pattern at positions 288 and 293, with V288/G293 and Q288/Q293 haplotypes co-circulating in our sample, is consistent with the action of positive selection at these sites, as demonstrated by dN/dS analysis in recent studies [16,30]. Whether these substitutions alter antigenicity, receptor affinity, or viral fitness remains to be determined experimentally. Of particular interest are the substitutions detected at residues 314 and 383. The emergence of lysine at position 314 in four isolates (16%) represents a novel finding, given that arginine is conserved at this position in all previously reported avian and non-avian GyVg1 sequences, including the Brazilian and Chinese reference strains [36]. Similarly, the serine substitution at position 383 in isolate SD2024-23 introduces a polar uncharged residue into a region otherwise dominated by proline or glutamine. Position 383 lies within the C-terminal hypervariable cluster (383–419), which has been implicated in antigenic variation; thus, the 383S substitution may have implications for immune evasion or capsid stability. Future studies should employ site-directed mutagenesis and reverse genetics to dissect the functional consequences of the 314K and 383S mutations, particularly their effects on viral replication kinetics and neutralization sensitivity.

Several limitations of this study should be acknowledged. First, the clinical samples were collected exclusively from Shandong Province; therefore, the prevalence data and genetic diversity reported here may not be fully representative of other geographic regions in China or other countries. Second, although our duplex real-time PCR assay has quantitative capability, we did not perform systematic viral load analysis, which would provide deeper insights into disease progression and viral interactions. Third, this study is based on molecular detection and lacks experimental pathogenicity data; thus, the pathogenic role of GyVg1 and its potential synergy with CAV remain to be confirmed through controlled infection trials. Future studies incorporating multi-regional sampling, longitudinal monitoring, and functional pathogenicity experiments are warranted to address these gaps.

In summary, the duplex TaqMan real-time PCR method developed in this study is an effective, sensitive, and specific tool for detecting CAV and GyVg1 simultaneously. The method’s application revealed a high co-infection rate of CAV and GyVg1. This, coupled with GyVg1’s genetic diversity and cross-species transmission characteristics, offers valuable insights for comprehending the epidemiological dynamics of these viruses. Additionally, it aids in formulating evidence-based prevention and control strategies.

## Figures and Tables

**Table 1:** Primer and probe sequences designed in this study.

No.	Primer Name	Primer Sequence (5′–3′)
1	GyVg1-F	CGCTTTGGCTTCTACCACAG
	GyVg1-R	TCTACGCGCATATCGAAATTTAC
	GyVg1-P	FAM-CACTCCACACACACACTCCCACCA-TAMRA
2	CAV-F	CGGATTGGTATCGCTGGAAT
	CAV-R	GTCCGCAGTTGCAGATCTTA
	CAV-P	VIC-ACAATCACTCTATCGCTGTGTGGCTG-BHQ1
3	GyVg1-1-F	GGGTACGTCACAGCCAATCAGAA
	GyVg1-1-R	CCCATAGCGTCCAGAGTCATTAG
4	GyVg1-2-F	TATTCCCGGAGGGGTAAATTTCGAT
	GyVg1-2-R	CCCCTGTCCCCGTGATGGAATGTTT
5	GyVg1-3-F	GCTTAGAAGACAGTAGCAGATGCT
	GyVg1-3-R	GTTGTATCTGTCCGTTGCAGTATG
6	GyVg1-4-F	AGCAGCCTGTACACAGAGA
	GyVg1-4-R	TCTTGCCTAAGCGGCAATTCA

Note: Primer/probe sets 1 and 2 were used for qPCR detection of GyVg1 and CAV, respectively; primers 3–6 amplified the complete genome of GyVg1, yielding fragments of 1207, 982, 455, and 446 bp.

**Table 2:** Reference sequence information of GyVg1 used for phylogenetic analysis in this study.

Isolate Name	Time	Location	Source	Accession Numb
Avian gyrovirus 2	2012	China	Chicken	JQ690763
HLJ1506-1	2015	China	Chicken	KX708506
NX1506-1	2015	China	Chicken	KX708508
JL1508	2015	China	Chicken	KX708511
BJ1509	2015	China	Chicken	KX708512
LN1511	2015	China	Chicken	KX708515
HE1511	2015	China	Chicken	KX708514
GS1512	2015	China	Chicken	KX708517
GZ1601	2016	China	Chicken	KX708518
JX1602	2016	China	Chicken	KX708519
HLJ1603-1	2016	China	Chicken	KX708520
33-SD201908	2019	China	Chicken	OQ116645
17CC0315	2019	China	Chicken	MK089244
GyG1-SDAU-1	2020	China	Chicken	OL448986
NC19-GyVg1-01	2023	China	Chicken	OL406403
32-IM201910	2023	China	Chicken	OQ116644
ZJ	2024	China	Chicken	OR355455
JS	2024	China	Chicken	OR355451
HN2401	2024	China	Chicken	PP827010
KGSM/M0313-2Li	1997	Japan	Chicken	LC716405
KGSM/N0313-5S	1997	Japan	Chicken	LC716407
Ave 3	2006	Brazil	Chicken	HM590588
RS-BR-15-2S	2015	Brazil	Chicken	MG846492
UP455/13b	2013	South Africa	Chicken	KF436510
S53-It	2014	Italy	Chicken	KU168250
915F06007FD	2009	France	Homo sapiens	FR823283
JQ690763	2012	China	Homo sapiens	JQ690763
AGV2-GXHG-32	2019	China	Dog	OK245349
DOG01	2021	China	Dog	OR921198
17CC0810	2017	China	Domestic cat	MK089246
G17	2014	Hungary	Mustela putorius furo	KJ452213
HB2018-S1	2018	China	Elaphe carinata	MK840982
HN2019-S1	2019	China	Silver pheasant	OK540280
HN2019-H1	2019	China	Hippopotamus	OK540281
HN2019-T1	2019	China	Tiger	OK540282

**Table 3:** Intra-assay and inter-assay reproducibility test results.

	Template Concentration (Copies/μL)	Intra-Assay Ct Mean ± SD	Intra-Assay CV (%)	Inter-Assay Ct Mean ± SD	Inter-Assay CV (%)
GyVg1	1 × 10^7^	18.31 ± 0.10	0.56	18.38 ± 0.22	1.18
	1 × 10^5^	25.05 ± 0.21	0.84	25.17 ± 0.34	1.35
	1 × 10^3^	31.92 ± 0.33	1.02	32.08 ± 0.75	2.34
CAV	1 × 10^7^	19.11 ± 0.09	0.48	19.20 ± 0.20	1.05
	1 × 10^5^	25.73 ± 0.18	0.71	25.88 ± 0.32	1.24
	1 × 10^3^	32.58 ± 0.31	0.95	32.70 ± 0.72	2.21

**Table 4:** Comparison of CAV and GyVg1 detection results between duplex real-time PCR and conventional PCR assays.

		Duplex Real-Time PCR			Conventional PCR		
		GyVg1			GyVg1		
		Positive	Negative	Total	Positive	Negative	Total
CAV	Positive	72	128	200	54	108	162
	Negative	5	206	211	8	241	249
	Total	77	334	411	62	349	411

**Table 5:** Sequence information obtained in this study.

No.	Isolate Name	Accession No.	Source	Length
1	SD2024-1	PX718866.1	broiler	2376 bp
2	SD2024-2	PX718867.1	broiler	2376 bp
3	SD2024-3	PX718868.1	broiler breed	2376 bp
4	SD2024-4	PX718869.1	broiler breed	2376 bp
5	SD2024-5	PX718870.1	layer	2376 bp
6	SD2024-6	PX718871.1	layer	2376 bp
7	SD2024-7	PX718872.1	broiler breed	2376 bp
8	SD2024-8	PX718873.1	broiler breed	2376 bp
9	SD2024-9	PX718874.1	broiler breed	2376 bp
10	SD2024-10	PX718875.1	broiler	2376 bp
11	SD2024-11	PX718876.1	broiler breed	2376 bp
12	SD2024-12	PX718877.1	broiler	2376 bp
13	SD2024-13	PX718878.1	broiler breed	2376 bp
14	SD2024-14	PX718879.1	broiler breed	2376 bp
15	SD2024-15	PX718880.1	broiler	2376 bp
16	SD2024-16	PX718881.1	broiler breed	2376 bp
17	SD2024-17	PX718882.1	broiler breed	2376 bp
18	SD2024-18	PX718883.1	layer	2376 bp
19	SD2024-19	PX718884.1	broiler breed	2376 bp
20	SD2024-20	PX718885.1	broiler	2376 bp
21	SD2024-21	PX718886.1	broiler breed	2376 bp
22	SD2024-22	PX718887.1	broiler breed	2376 bp
23	SD2024-23	PX718888.1	broiler breed	2376 bp
24	SD2024-24	PX718889.1	broiler	2376 bp
25	SD2024-25	PX718890.1	layer	2376 bp

**Table 6:** Mutations at different amino acid sites in GyVg1 VP1.

Stain	36	44	73	106	212	242	270	288	293	310	314	383	390	401	416	433	459
AGV2/China	G	R	L	S	K	R	S	Q	Q	E	R	P	I	V	L	W	N
AGV2-GXHG-32/China	G	K	L	S	K	R	S	V	G	E	R	P	I	V	L	R	N
Ave_3/Brazil	G	R	L	S	R	G	A	V	G	Q	R	Q	I	M	I	W	N
SD2024-1	G	R	L	S	K	R	S	V	G	E	R	P	I	V	L	W	N
SD2024-2	G	R	L	S	K	R	S	Q	Q	E	R	P	I	V	L	W	N
SD2024-3	G	R	L	S	K	R	S	V	G	E	R	P	I	V	L	W	N
SD2024-4	G	R	L	S	K	R	S	Q	Q	E	R	P	I	V	L	W	N
SD2024-5	G	R	L	S	K	R	S	V	G	E	K	P	I	V	L	W	N
SD2024-6	G	R	L	S	K	R	S	Q	Q	E	K	P	I	V	L	W	N
SD2024-7	G	R	L	S	K	R	S	V	G	E	R	P	I	V	L	W	N
SD2024-8	G	R	L	S	K	R	S	V	G	E	R	P	I	V	L	W	N
SD2024-9	G	R	L	S	K	R	S	V	G	E	R	P	I	V	L	W	N
SD2024-10	G	R	L	S	K	R	S	V	G	E	R	P	I	V	L	W	N
SD2024-11	G	R	L	S	K	R	S	Q	Q	E	K	P	I	V	L	W	N
SD2024-12	G	R	L	S	K	R	S	V	G	E	K	P	I	V	L	W	N
SD2024-13	G	R	L	S	K	R	S	V	G	E	R	P	I	V	L	W	N
SD2024-14	G	R	L	S	K	R	S	V	G	E	R	P	I	V	L	W	N
SD2024-15	G	R	L	S	K	R	S	V	G	E	R	P	I	V	L	W	N
SD2024-16	G	R	L	S	K	R	S	Q	E	E	R	P	I	V	L	W	N
SD2024-17	G	R	L	S	K	R	S	V	G	E	R	P	I	V	L	W	N
SD2024-18	G	R	L	S	K	R	S	Q	Q	E	K	P	I	V	L	W	N
SD2024-19	G	R	L	S	K	R	S	V	G	E	R	P	I	V	L	W	N
SD2024-20	G	R	L	S	K	R	S	V	G	E	R	P	I	V	L	W	N
SD2024-21	G	R	L	S	K	R	S	Q	Q	E	R	P	I	V	L	W	N
SD2024-22	G	R	L	S	K	R	S	Q	Q	E	R	P	I	V	L	W	N
SD2024-23	G	R	L	S	K	R	S	Q	Q	E	R	S	I	V	L	W	N
SD2024-24	G	R	L	S	K	R	S	Q	Q	E	R	P	I	V	L	W	N
SD2024-25	G	R	L	S	K	R	S	V	G	E	R	P	I	V	L	W	N

## Data Availability

Data is contained within the article or Supplementary Material.

## Supplementary Materials

The following supporting information can be downloaded at: https://www.mdpi.com/article/10.3390/v18080859/s1, Supplementary Table S1: Detailed information of 411 clinical samples collected from Shandong Province, China, in 2024; Supplementary Figure S1: Geographic distribution map of sample origins in this study.

## References

[1] Zhang S., Yuan S., Yan T., Li G., Hao X., Zhou D., Li R., Li Y., Cheng Z. (2022). Serological investigation of Gyrovirus homsa1 infections in chickens in China. BMC Vet. Res..

[2] Rosario K., Breitbart M., Harrach B., Segales J., Delwart E., Biagini P., Varsani A. (2017). Revisiting the taxonomy of the family Circoviridae: Establishment of the genus Cyclovirus and removal of the genus Gyrovirus. Arch. Virol..

[3] Schat K.A. (2009). Chicken anemia virus. TT Viruses.

[4] Duan Y., Gao C., Cao W., Yang X., Zuo M., Liang X., Yang Y., Fang X., Fan K., Tan L. (2025). Current Knowledge on the Diagnostic Methods, Epidemiological Characteristics and Antiviral Strategies of Chicken Anemia Virus. Vet. Sci..

[5] Gimeno I.M., Schat K.A. (2018). Virus-Induced Immunosuppression in Chickens. Avian Dis..

[6] Zhang Y., Xie Z., Xie Z., Xie L., Li M., Yan M., Wu A., Zhang M., Fan Q., Zeng T. (2026). Molecular Characterization of Emerging Gyrovirus galga 1 from Poultry Markets of Guangxi, China. Int. J. Mol. Sci..

[7] Cibulski S., Weber M.N., de Sales Lima F.E., Lima D.A., Fernandes Dos Santos H., Teixeira T.F., Varela A.P.M., Tochetto C., Mayer F.Q., Roehe P.M. (2020). Viral metagenomics in Brazilian Pekin ducks identifies two gyrovirus, including a new species, and the potentially pathogenic duck circovirus. Virology.

[8] Yan T., Zhao M., Sun Y., Zhang S., Zhang X., Liu Q., Li Y., Cheng Z. (2023). Molecular evolution analysis of three species gyroviruses in China from 2018 to 2019. Virus Res..

[9] Eltahir Y.M., Qian K., Jin W., Wang P., Qin A. (2011). Molecular epidemiology of chicken anemia virus in commercial farms in China. Virol. J..

[10] Di Francesco A., Quaglia G., Salvatore D., Sakhria S., Catelli E., Bessoussa G., Kaboudi K., Ben Chehida N., Lupini C. (2021). Occurrence of Chicken Infectious Anemia Virus in Industrial and Backyard Tunisian Broilers: Preliminary Results. Animals.

[11] Adair B.M. (2000). Immunopathogenesis of chicken anemia virus infection. Dev. Comp. Immunol..

[12] Yan T., Song Y., Zhang D., Wang Z., Li R., Zhang X., Xiang B., Yang L., Lang F., Chu D. (2025). Chicken anemia virus inhibits hematopoiesis and development of chicken embryo. Poult. Sci..

[13] Fatoba A.J., Adeleke M.A. (2019). Chicken anemia virus: A deadly pathogen of poultry. Acta Virol..

[14] Chen S., Xu H., Li W., Nie Y., Xie Q., Chen W. (2024). Deciphering Immune Modulation in Chickens Co-Infected with ALV-J and CIAV: A Transcriptomic Approach. Microorganisms.

[15] Rijsewijk F.A., Dos Santos H.F., Teixeira T.F., Cibulski S.P., Varela A.P., Dezen D., Franco A.C., Roehe P.M. (2011). Discovery of a genome of a distant relative of chicken anemia virus reveals a new member of the genus Gyrovirus. Arch. Virol..

[16] Zhang F., Xie Q., Yang Q., Luo Y., Wan P., Wu C., Tu L., Chen J., Kang Z. (2024). Prevalence and phylogenetic analysis of Gyrovirus galga 1 in southern China from 2020 to 2022. Poult. Sci..

[17] Tran G.T.H., Huynh L.T.M., Dong H.V., Rattanasrisomporn A., Kayan A., Bui D.A.T., Rattanasrisomporn J. (2024). Detection and Molecular Characterization of Gyrovirus Galga 1 in Chickens in Northern Vietnam Reveals Evidence of Recombination. Animals.

[18] Ji J., Yu Z., Cui H., Xu X., Ma K., Leng C., Zhang X., Yao L., Kan Y., Bi Y. (2022). Molecular characterization of the Gyrovirus galga 1 strain detected in various zoo animals: The first report from China. Microbes Infect..

[19] Turan T., Isidan H., Duran-Yelken S., Atasoy M.O., Ozbek R., El Naggar R.F., Rohaim M.A. (2025). Genetic and Statistical Study of Anelloviruses and Gyroviruses in Diarrheic Cats and Their Co-Occurrence Patterns. Viruses.

[20] Mase M., Yamamoto Y., Iseki H., Tanikawa T., Kurokawa A. (2022). Detection of Gyrovirus galga 1 in Cryopreserved Organs from Two Commercial Broiler Flocks in Japan. Viruses.

[21] Coronado L., Munoz-Aguilera A., Wang M., Munoz I., Riquelme C., Heredia S., Stepniewska K., Gallardo C., Ganges L. (2025). Simultaneous Detection of Classical and African Swine Fever Viruses by Duplex Taqman Real-Time PCR Assay in Pigs Infected with Both Diseases. Pathogens.

[22] Liu Z., Jiang Q., Yang Y., Qi R., Gu H., Chen M., Feng K., Jia H., Kang H., Liu J. (2025). Establishment of one-step duplex TaqMan real-time PCR for detection of feline coronavirus and panleukopenia virus. Appl. Microbiol. Biotechnol..

[23] Varela A.P.M., Dos Santos H.F., Cibulski S.P., Scheffer C.M., Schmidt C., Sales L.F., Rijsewijk F.A.M., Roehe P.M. (2014). Chicken anemia virus and avian gyrovirus 2 as contaminants in poultry vaccines. Biologicals.

[24] Kaffashi A., Pagel C.N., Noormohammadi A.H., Browning G.F. (2015). Evidence of apoptosis induced by viral protein 2 of chicken anaemia virus. Arch. Virol..

[25] Fujiwara A., Horii W., Sano J., Kodama T., Kato A., Shibuya K., Saitoh T. (2024). Invasion of Chicken Anemia Virus in Specific-Pathogen-Free Chicken Flocks and Its Successful Elimination from the Colony. Vet. Sci..

[26] Xu S., Zhang Z., Xu X., Ji J., Yao L., Kan Y., Xie Q., Bi Y. (2023). Molecular Characteristics of Chicken Infectious Anemia Virus in Central and Eastern China from 2020 to 2022. Animals.

[27] Li J., Lou Y., Li P., Wang T., Lv Z., Guo Z., Geng N., Meng F., Liu S., Li N. (2023). Retrospective Investigation and Genetic Variation Analysis of Chicken Infectious Anemia in Shandong Province, 2020–2022. Vet. Sci..

[28] Snoeck C.J., Komoyo G.F., Mbee B.P., Nakoune E., Le Faou A., Okwen M.P., Muller C.P. (2012). Epidemiology of chicken anemia virus in Central African Republic and Cameroon. Virol. J..

[29] Wu Y., Su Y., Cao H., Wang Y., Zheng S.J., Gao L. (2026). Chicken infectious Anemia virus impairs vaccination-induced antibody responses by suppressing CD40L expression. Vaccine.

[30] Zhang Z., Xu X., Li D., Liu F., Wang L., Yao L., Ji J., Xie Q., Bi Y. (2025). Genetic and recombination analysis of GyVg1 varients from companion animals in central and northwest China. Front. Vet. Sci..

[31] Zhang Z., Xu X., Li D., Yao L., Ji J., Xie Q., Bi Y. (2025). First report of inter-species recombinant Gyroviruses in Chinese urban companion animals with cross-species transmission risks. Front. Vet. Sci..

[32] Yang M., Yang Q., Bi X., Shi H., Yang J., Cheng X., Yan T., Zhang H., Cheng Z. (2023). The Synergy of Chicken Anemia Virus and Gyrovirus Homsa 1 in Chickens. Viruses.

[33] Xu S., Man Y., Yu Z., Xu X., Ji J., Kan Y., Bi Y., Xie Q., Yao L. (2024). Molecular analysis of Gyrovirus galga1 variants identified from the sera of dogs and cats in China. Vet. Q..

[34] Liu Y., Lv Q., Li Y., Yu Z., Huang H., Lan T., Wang W., Cao L., Shi Y., Sun W. (2022). Cross-species transmission potential of chicken anemia virus and avian gyrovirus 2. Infect. Genet. Evol..

[35] Zhang Z., Man Y., Xu X., Wang Y., Ji J., Yao L., Bi Y., Xie Q. (2024). Genetic heterogeneity and potential recombination across hosts of Gyrovirus galga1 in central and eastern China during 2021 to 2024. Poult. Sci..

[36] Tran G.T.H., Rattanasrisompor A., Bui D.A.T., Dong H.V., Rattanasrisomporn J. (2026). Molecular characterization of Gyrovirus galga 1 in domestic dogs in the north of Vietnam indicates the presence of recombination events. Int. J. Mol. Sci..

