# Supplementary material for: Establishment and Application of a Duplex TaqMan Real-Time PCR Assay for Simultaneous Detection of Chicken Anemia Virus (CAV) and Gyrovirus Galga 1 (GyVg1)
Source: Viruses. 2026 Aug 6;18(8):859. doi: 10.3390/v18080859 (PMC13517485; doi:10.3390/v18080859)
Supplement: Supplementary file 1 [file viruses-18-00859-s001.zip › viruses-4452924-supplementary.pdf]

Supplementary Table S1. Detailed information of 411 clinical samples collected from Shandong Province, China, in 2024.

City	No. Of samples	Broilers (1-4 wks)	Broiler breeders (8-20 wks)	Layers	Cloacal swabs	Tissue samples
Tai'an	98	58	22	18	75	23
Linyi	76	48	16	12	58	18
Weifang	65	42	14	9	48	17
Jining	54	35	11	8	42	12
Dezhou	48	28	10	10	38	10
Qingdao	18	12	4	2	13	5
Jinan	12	8	3	1	10	2
Zibo	10	7	3	0	8	2
Yantai	9	6	2	1	7	2
Weihai	8	5	2	1	6	2
Rizhao	7	4	1	2	6	1
Liaocheng	6	3	1	2	5	1
Total	411	256	89	66	312	99

Note: Tissue samples included thymus (n=42), bursa of Fabricius (n=35), and bone marrow (n=22). All birds exhibited clinical signs consistent with infectious anemia or were from flocks with suspected immunosuppression.

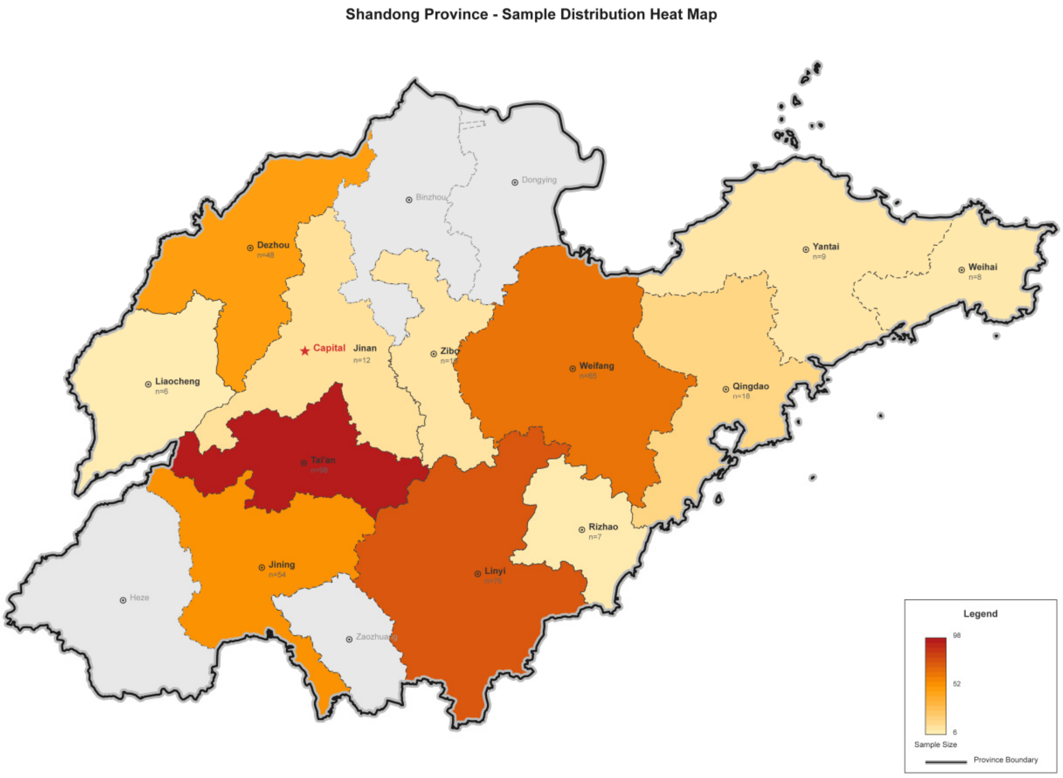

Supplementary Figure S1. Geographic distribution map of sample origins in this study.
